# Longitudinal Associations Between Anxiety and Depressive Symptoms in Adolescence, Early Adulthood, and Old Age: Cross-Lagged Panel Network Analyses

**DOI:** 10.1155/da/6205475

**Published:** 2024-11-29

**Authors:** Shoushi Wang, Zh Yeng Chong, Chunyang Zhang, Wei Xu

**Affiliations:** ^1^Beijing Key Laboratory of Applied Experimental Psychology, National Demonstration Center for Experimental Psychology Education (Beijing Normal University), Faculty of Psychology, Beijing Normal University, Beijing, China; ^2^The Aviation Safety Research Institute, China Academy of Civil Aviation Science and Technology, Beijing, China

**Keywords:** anxiety, cross-lagged panel networks, depression

## Abstract

**Background:** Depression and anxiety are among the most prevalent psychiatric disorders worldwide, affecting individuals of all ages. The co-occurrence of these disorders often exacerbates their negative health impacts, underscoring the necessity of understanding their comorbid mechanisms.

**Methods:** This study employed cross-lagged panel networks (CLPNs) to explore the longitudinal associations between depression and anxiety symptoms across three age groups and to compare the respective symptom networks. CLPNs were constructed through cross-temporal associations between different symptoms, reflecting both the pattern of interaction and the significance of specific symptoms in comorbidity. The sample consisted of 1258 adolescents (aged 13–19 years, *M* = 15.98), 1118 college students (aged 17–24 years, *M* = 19.94), and 548 older adults (aged 60–101 years, *M* = 85.19) from China. Depression and anxiety symptoms were assessed using the subscales of the Depression, Anxiety, and Stress Scale Short Version (DASS-21) at two time points over a 6-month period during 2020–2021.

**Results:** The findings revealed that the prevalence of depression and anxiety in adolescents, college students, and older adults was 25.9%/46.6%, 53.7%/61.5%, and 7.2%/22.5%, respectively. The network structure varied across age groups: adolescents and college students exhibiting a tight interconnection between depression and anxiety symptoms, while older adults showed stronger small-world network characteristics. A key finding across all age groups was the central role of irrational fear. In addition, somatic anxiety symptoms frequently emerged as outcomes of other psychological symptoms.

**Conclusion:** Depression and anxiety are more pronounced in college students compared to adolescents and older adults. Comparisons of the overall network structure provide insights into the lifelong trajectories of depression and anxiety symptom networks. The centrality of irrational fears and somatization symptoms is emphasized. These results offer guidance for more targeted clinical interventions.

## 1. Introduction

Depression and anxiety represent the most pervasive mental health challenges globally ([1], p. 40). Mental disorders are recognized as significant contributors to the burden of health-related issues, with depression and anxiety disorders being among the foremost contributors to this burden ([1], p. 50). These disorders pose a significant threat across all demographics, a situation exacerbated by the COVID-19 pandemic [2]. Researchers estimate that as a result of the pandemic, there will be 54 million additional cases of major depression globally, bringing the overall prevalence to 3.1%, alongside an estimated additional 83.0 million cases of anxiety disorders globally, raising the overall prevalence to 4.8% [2]. Adolescents, in particular, exhibit high susceptibility to emotional disturbances, with reported prevalence rates of depression symptoms and anxiety symptoms at 25.2% and 20.5%, respectively [3]. A large-scale meta-analysis of 192 epidemiological studies showed that one-third, half, and 62.5% of individuals experience their first mental disorders before the ages of 14, 18, and 25, with peak/median ages being 15.5/18 years [4]. This suggests that mental disorders originate in early developmental stages of life and peak in mid-to-late adolescence, underscoring the importance of studying psychological health issues among adolescents and college students in early adulthood. In addition to these, the mental well-being of older adults garners attention amidst global aging trends. Meta-analytic evidence suggests a 13.3% global prevalence of major depression among the elderly [5], with individuals over 80 facing heightened mental health risks [6, 7]. Other results show that the lifetime prevalence of anxiety in older adults is as high as 14.9% [8]. Some studies have predicted that the highest increase in the prevalence of mental disorders will be in the elderly, and that this increase is largely due to population aging [9]. For the above reasons, this study considered that the mental health of adolescents, college students in early adulthood, and older adult populations deserves additional attention.

This incidence of co-occurring depression and anxiety is notably significant. Studies indicate that 51% of individuals diagnosed with severe depression also receive an anxiety diagnosis within the same year [10]. Simultaneously, individuals with moderate levels of depression and anxiety are more prone to functional impairments and suicidal ideation [11, 12]. Understanding the interplay between anxiety and depression is crucial. For instance, a birth cohort study in Dunedin indicated that in individuals aged 11–32, anxiety preceded or occurred simultaneously with depression in 37% of cases, while depression preceded or coincided with anxiety in 32% of instances [13]. In addition, a 9-year longitudinal study found that 6% of older participants developed comorbid depression and anxiety disorder, leading to significant functional decline [14]. Given these findings, the current study aims to investigate and delineate the patterns of comorbid depression and anxiety across three life stages: adolescents, early adulthood (e.g., college students), and later life.

The tripartite model, proposed by Clark and Watson [15], delineates the underlying mechanisms of comorbidity between anxiety and depression. It states that negative affect (encompassing emotions such as anger, guilt, fear, sadness, and worry) is a common element in both disorders, serving as a foundation for their comorbidity. In contrast, physiological hyperexcitability (manifested through symptoms like somatic tension, shortness of breath, dizziness, and dry mouth) is specific to anxiety, while low positive affect (characterized by fatigue, slowness, tiredness) is typical of depression [15, 16]. Empirical studies have actively tested this theory. For example, Teachman, Siedlecki, and Magee [17] validated the tripartite model across a broad age range (18–93 years), whereas Olino et al. [18] demonstrated its superiority over a unidimensional internalizing model in individuals under 30 years. However, some studies challenge the model, arguing that physiological hyperexcitability may not clearly differentiate between anxiety and depression. Instead, physiological hyperexcitability might act as a common element in both conditions [19, 20]. The divergent findings [16, 21] suggest variability within the model's factors, indicating a need for a more nuanced analysis of comorbidity components.

Traditionally, comorbidity research has employed latent variable models, assuming that comorbidity arises from the relationship between two underlying factors [22–24]. However, critiques of this approach [25] advocate for direct causal relationships among symptoms across disorders, challenging the common-cause assumption. For example, a study found that difficulty concentrating played a central role in the persistence of anxiety and mood disorders symptoms [26]. This criticism has led to the proposal of network analysis as a method to elucidate mental disorder mechanisms [25, 27]. Network analysis quantifies the complex associations between symptoms, depicting diseases as networks of interdependent symptoms, or “bridge symptoms,” which foster psychiatric comorbidity [28]. Previous studies have analyzed the networks of anxiety and depression comorbidities across age groups, yielding varied results [29–33]. A recent meta-analysis of 33 cross-sectional studies identified sad mood, uncontrollable worry, and worrying too much as the most central symptoms, while sad mood, restlessness, and motor disturbance were the most frequent bridge centrality symptoms [34]. However, cross-sectional network analysis, although useful for identifying core nodes, does not reveal the chronological order of symptom onset or directional interactions. To address this limitation, cross-lagged panel network (CLPN) has been introduced [35]. CLPN requires longitudinal data, involving repeated measurements of the same individuals over multiple time points, enabling the examination of temporal dynamics and directional relationships between variables. By modeling interactions at various time intervals, CLPN provides insights into the reciprocal relationships and potential causal pathways between psychological constructs.

Two studies have investigated the predictive relationship between depression and anxiety symptoms using CLPN. Zhang, Huang, and Xu [33] addressed depression and generalized anxiety symptoms in adolescents, finding a close association between the two, with generalized anxiety symptoms predicting depressive symptoms. Difficulty relaxing had the highest expected impact. Zou et al. [36] found bidirectional prediction of depression and anxiety symptoms among college students. Depression predicted anxiety symptoms more frequently, and the predictive strength from depression to anxiety was greater. In this network, irritability, guilt, and sad mood emerged as key symptoms, suggesting that symptom networks for depression and anxiety may differ across age. McElory et al. [30] assessed the relationship between depression and anxiety in children to early adolescents, observing that network connectivity increased with age. Feelings of anxiety/fear and unhappiness/sadness were the most central symptoms throughout development, suggesting changes in network structure over time. However, no studies have yet analyzed CLPN for anxiety and depressive symptoms in older adults. Cross-sectional studies have identified uncontrollable worry, sad mood, and difficulty relaxing as important symptoms in comorbidity of depression and anxiety [37, 38], suggesting that these symptoms may be central across all age groups.

In summary, studies examined both cross-sectional network and CLPN for depression and anxiety, yet the results remain inconclusive [34]. Furthermore, no studies have systematically compared longitudinal network differences across age groups, and studies focusing on older adults are particularly scarce. This gap limits the generalizability of findings across the lifespan. The present study examines similarities and differences in symptom networks across age groups, providing preliminary evidence for the lifelong development of depression and anxiety comorbidities. From a practical standpoint, identifying core symptoms pivotal in the development of these disorders and their comorbidities across age groups could enhance the specificity and effectiveness of clinical interventions. In addition, employing longitudinal data to construct CLPN models could afford a more nuanced understanding of the temporal effects and causal relationships among symptoms.

In light of these theoretical and practical considerations, this study leverages longitudinal data over a 6-month period for three distinct age groups: adolescents, college students, and older adults. The aim is to explore the longitudinal relationships of symptoms across age groups. Specifically, the study focuses on two primary objectives: (1) analyzing the longitudinal symptom networks of anxiety and depression to explore core bridge symptoms connecting the two disorders across the age spectrum and (2) evaluating the variations in these networks among different age groups, thereby contributing to a more comprehensive understanding of depression and anxiety comorbidity throughout the lifespan. Due to the mixed results of previous studies and the lack of established theories examining age differences in depression and anxiety comorbidity, the present study took an exploratory approach, and as such, we did not propose specific hypotheses.

## 2. Method

### 2.1. Participants and Procedures

This study utilized a longitudinal dataset spanning 6 months, collected during 2020−2021[39]. The sample was selected using a convenience sampling method and included adolescents, college students, and older adults. Data collection for adolescents and college students was conducted online, while older adults participated through paper-and-pencil tests, facilitated by social workers. Adolescents were recruited from two secondary schools in Tiemenguan, Xinjiang, and Dingxi, Gansu. Baseline data (T1) were collected in September 2020, followed by a second round of data connection (T2) in February 2021. The college student group, consisting of participats from two universities in Lanzhou, Gansu, was surveyed in April 2021 (T1) and October 2021 (T2). Questionnaires were distributed to adolescents and college students through web links sent via their school. The older adult group consisted of community-dwelling individuals in Nanjing, Jiangsu. Community workers were trained by the researcher, after which they visited participants' homes to administer the questionnaire one-on-one. Data for the older adult group were collected in September 2020 (T1) and March 2021 (T2). Throughout the data collection periods, CODIV-19 prevention and control measures were in place, including daily nucleic acid testing and wearing masks. No significant local outbreaks or lockdowns occurred during this time.

For sample size, there are no well-established studies that give recommended sample sizes for CLPN. Wysocki et al. [35] note that a sample size must exceed the total number of variables across all measurement occasions (in this study, 36 variables) to ensure the covariance matrix to be positively determined. The sample size in this study exceeded this requirement. To verify whether the difference in sample size significantly affects the network structure, we used bootstrap to verify the stability of edges and centrality.

### 2.2. Measures

Participants provided demographic information and completed assessments for depression and anxiety using the Depression, Anxiety, and Stress Scale Short Version (DASS-21) developed by Lovibond and Lovibond [40]. Moussa, Lovibond, and Laube [41] translated the DASS-21 into traditional Chinese, while Gong et al. [42] translated it into simplified Chinese. The DASS-21 employs a 4-point Likert scale ranging from 0 (“does not meet”) to 3 (“always meets”). Items 3, 5, 10, 13, 16, 17, and 21 constitute the depression subscale. Items 2, 4, 7, 9, 15, 19, and 20 comprise the anxiety subscale. According to Lovibond and Lovibond [40], the DASS-21 subscale scores are summed and multiplied by 2 to obtain the total score. A depression score of 0–9 is considered normal, while 10 or above indicates mild or higher depression. For anxiety, a total score of 7 or less is normal, while 8 or higher indicates mild or above anxiety [40]. These cutoffs were used in the prevalence calculations for this study.

The DASS-21 has demonstrated reliability in various populations, including adolescents, college students, and older adults [43, 44]. It has also been widely used in Chinese contexts [42, 45, 46]. The reliability reports for each administration of the specific dimensions are shown in Table 1.

### 2.3. Data Analysis

CLPNs were generated using the methodology outlined by Wysocki et al. [35] in R 4.0.1. These networks were constructed to analyze autoregressive and cross-lagged associations between symptoms at T1 and T2. Autoregressive associations indicate the persistence of a symptom (e.g., “anhedonia”) from T1 to T2, while cross-lagged associations reflect the predictive relationship between a symptom at T1 and another at T2 (e.g., “anhedonia” leading to “difficulty initiating actions”). Covariate, such as age and gender, were controlled according to the method proposed by Funkhouser et al. [47]. This method involves incorporating both T1 variables and covariates as explanatory variables into Least Absolute Shrinkage and Selection Operator (LASSO) regressions. Thus, it ensured that predictions of T2 variables considered not only T1 variables but also the influence of covariates.

Both autoregression and cross-lagged estimation were employed, considering all variables within the analysis. Given the limited sample size, fivefold cross-validation and LASSO regularization were applied, effectively nullifying minor associations. In the binomial regression framework, exponentiation of coefficients facilitated the representation of odds ratios (ORs), with a value of 1 indicating no association. In addition, network influence was quantified through the computation of in- and out-expected influence (in-EI and out-EI), summarizing the directional associations between a specific symptom and all other symptoms. The concept of bridge-expected impact (bridge-EI) was introduced to encapsulate the association of symptoms across different disorder communities. The network's robustness was scrutinized through bootstrapping, conducting 1000 iterations using the bootnet package [48]. This process encompassed nonparametric bootstrapping for edge stability and case-drop bootstrapping for centrality stability assessments. Edge stability was evaluated by determining the frequency of edges that maintained or crossed a coefficient value of 1, acknowledging that the 95% confidence interval may not accurately reflect the true interval due to regularization-induced contraction. A case deletion bootstrap procedure was employed to investigate central stability using the correlation stability coefficient (CS-C), which indicates the maximum proportion of cases that can be deleted from the sample without significantly affecting the centrality indicator. According to Epskamp, Borsboom, and Fried [48], the CS-C of at least 0.25 is required for centrality measures to be considered reliable.

To compare the differences between networks across different age groups, initial analysis involved delineating network characteristics using the qgraph package [49]. This exploration induced metrics such as transitivity, average path length (APL), and the small-world index [50]. Transitivity quantified the network's tendency for clustering, while APL assessed the efficiency of information or influence propagation within the network. The small-world index provided a comparative measure of the network's resemblance to a small-world network, characterized by substantial clustering and minimal APL [50]. Additionally, random networks models were generated to establish baselines for comparison. Comparative analysis of CLPNs across the adolescences, college students, and elderly cohorts involved the use of the Jaccard similarity index. This index calculated the proportion of edges that were consistently present, with the same directional sign, across the compared networks [47, 51], thereby offering insights into the structural commonalities and differences between the networks.

### 2.4. Missing Data

Little's test was used to assess whether the missing data were missing completely at random (MCAR). To preserve as much information as possible, the expectation–maximization (EM) method was applied to fill in missing data for the adolescent and college student group, as well as part of the older adult group (due to relocation or hospitalization). The EM method is considered effective for providing unbiased parameter estimates under both missing at random (MAR) and MCAR conditions [52]. Since some older adults were lost due to death, their data were not classified as missing [53] and were excluded from the CLPN analysis. Attrition analysis was conducted using a *t*-test to compare whether baseline anxiety, depression, and age between participants with baseline data only and those with data from both time points significant differences existed.

## 3. Result

### 3.1. Participant Characteristics

A total of 1258 junior high school students in grades 1–3 (*M*_age_ = 15.98, standard deviation [SD] = 0.97) participated in the baseline measurements, with 526 from Xinjiang and 618 were from Gansu. Of the adolescent participants, 45.8% were male and 54.1% were female, with gender data missing for two individuals. A posttest was completely by 1154 adolescents (91.6%, *M*_age_ = 16.07, SD = 1.16), with 45.1% being male. The group of college students (*M*_age_ = 19.94, SD = 1.33) included 1118 participants. Of these, 58.9% were male and 41.1% were female. At T2, 907 university students participated (81.13%, *M*_age_ = 20.33, SD = 1.10), with 57.1% male. This sample covered all grade levels and included students from 12 different majors such as education, engineering, economics, and linguistics. Reasons for data loss in the adolescent and college student population included temporary illness, leave of absence, and withdrawal. The older adult sample included 548 community-dwelling individuals (*M*_age_ = 85.19, SD = 8.75) in Nanjing, with 46.5% male and 53.5% female participants. A total of 457 older adults (83.40%, *M*_age_ = 85.00, SD = 7.98, 46.4% male) participated in T2, with 5 individuals (1%) withdrawing due to relocation, incapacitation, or hospitalization, and 86 (15.69%) lost due to death. The final network analysis included 462 older people.

### 3.2. Attrition Analysis

The Little's MCAR test indicate that missing data in the older adults and adolescent groups fell under the MCAR assumption, while the college student data did not. Among adolescent, those who dropped out were significantly older than those who remained (*t* = 4.549, *p* < 0.001), with no significant differences in baseline depression and anxiety levels. Withdrawn college students had significantly higher baseline depression and anxiety than those who completed both time points (*t*_depression_ = 4.35, *p* < 0.001; *t*_anxiety_ = 3.15, *p*=0.002), but there were no age differences. Among older adults, there were no significant differences in baseline depression, anxiety, or age between those who withdrew and those who continued. Detailed dropout analysis results are presented in Table S1.

### 3.3. Descriptive Analyses

Descriptive statistics for depression and anxiety scores and prevalence across each time period for all groups are shown in Table 2. College students exhibited the highest prevalence of depression and anxiety, with rates ranging from 51.8% to 55.60% for depression and 61.00% to 61.90% for anxiety. Adolescents followed with a prevalence of 21.60% to 30.10% for depression and 39.70% to 53.40% for anxiety. Older adults had the lowest prevalence, with depression rates ranging from 9.10% to 4.70% and anxiety rates 24.60% to 17.80%.

### 3.4. Accuracy and Stability of Network Parameters

The bootstrap analyses of edge weights demonstrated that the networks for all three age groups provided reliable edge estimates, as shown in Figure S1–S3. The centrality stability analysis showed that in the adolescent cross-lagged networks, in-EI displayed a stability of 0.36, exceeding the 0.25 threshold. However, out-EI and bridge-EI exhibited CS-C values of 0.21 each, failing below the threshold. For college students, both in-EI (0.36) and out-EI (0.28) exceeded the threshold, whereas bridge-EI (0.21) remained below it. In contrast, the elderly cross-lagged networks showed robust stability across all metrics, with in-EI, out-EI, and bridge-EI obtaining CS-C values of 0.60, 0.44, and 0.44, respectively, all of which exceed the minimum required threshold. Detailed centrality stability results are shown in Figure S4–S6. Therefore, centrality induced from networks that satisfy the stability criteria are the only ones considered for interpretation in this study.

### 3.5. Network Edges

**Figure 1:**
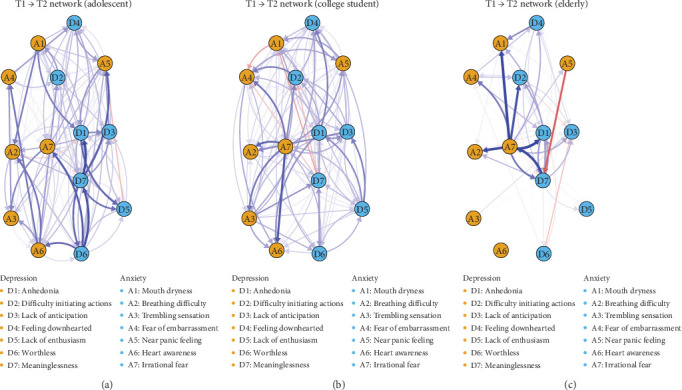
The T1–T2 cross-lagged panel networks for three age groups. *Note:* The cross-lagged panel networks for adolescents (a), college students (b), and old adults (c). The arrows represent unique longitudinal relationships. The blue edges indicate positive relationships (i.e., odds ratios greater than 1), and the red edges indicate negative relationships (i.e., odds ratios less than 1). Edge thickness represents the strength of the odds ratio such that thicker edges represent stronger relations. Autoregressive edges and covariates were excluded from the plot to ease visual interpretation.

Figure 1 illustrates the directed networks of CLPN results for each age group, with arrows directionality representing the longitudinal relationship between symptoms from T1 to T2, controlling for covariates such as gender and age. Each network graph uses the average layout of the three populations and the same maximum of the edge weights to facilitate visual comparisons across networks. In these figures, blue edges denote positive relationships (OR greater than 1), and red edges indicate negative relationships (OR less than 1). The edge thickness correlates with the strength of the OR, where thicker edges signify more robust relationships. Autoregressive edges, which are inherently present in each group and substantially stronger than cross-lagged edges, are omitted from Figure 1 to enhance visual clarity. The results of the autoregression are presented in Figure S7 of the Supporting Information.

In the adolescent CLPN, the most pronounced relationship is observed from meaninglessness (D7) to anhedonia (D1), with an OR of 1.18, making it significantly stronger than 60.7% of other edges. The most prominent bridge edges, linking symptoms across different domains, are from worthless (D6) to irrational fear (A7; OR = 1.14) and from meaninglessness (D7) to panic (A5; OR = 1.14). For college students, the strongest edge is from irrational fear (A7) to heart awareness (A6; OR = 1.16), differing significantly from 44.8% of the network's edges. The most significant bridge connection is observed between difficulty initiating actions (D2) and fear of embarrassment (A4; OR = 1.11). Within the elderly group, the three strongest edges are from irrational fear (A7) to anhedonia (D1; OR = 1.32); mouth dryness (A1; OR = 1.32); and breathing difficulty (A2; OR = 1.30). These edges are significantly higher than 90.3%, 88.2%, and 89.8%, respectively. The elderly network's most significant bridge edge is from irrational fear (A7) to anhedonia (D1). The specific results of the marginal difference test are presented in Figures S8–S10.

### 3.6. Network Centrality

**Figure 2:**
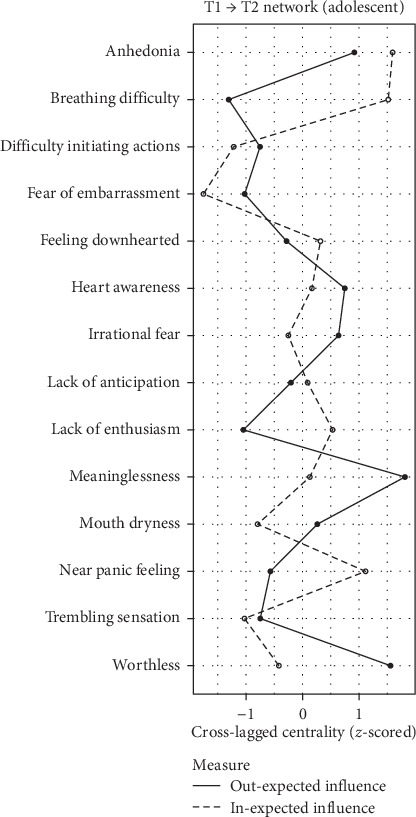
Symptom centrality estimates in the network of adolescent. *Note:* Larger values reflect greater centrality.

**Figure 3:**
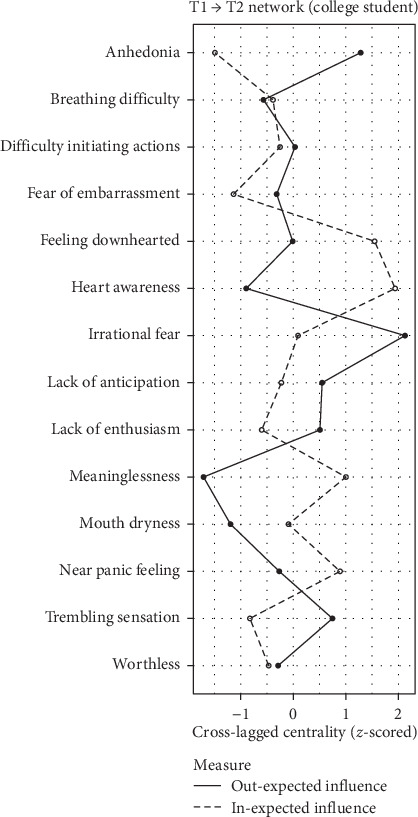
Symptom centrality estimates in the network of college students. *Note:* Larger values reflect greater centrality.

**Figure 4:**
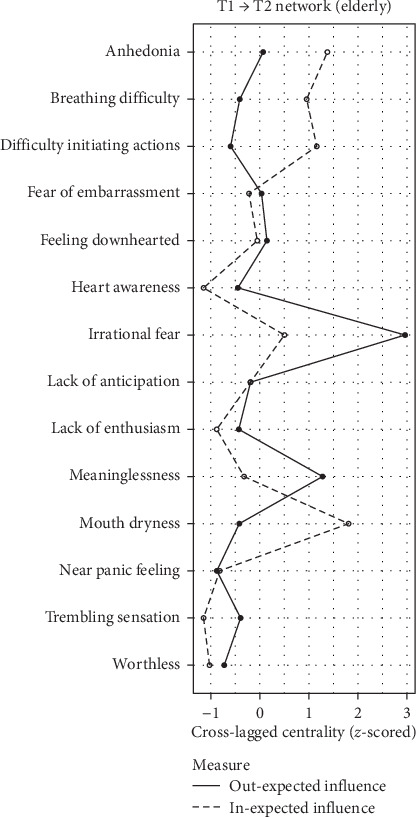
Symptom centrality estimates in the network of elderly. *Note:* Larger values reflect greater centrality.

Symptom centrality estimates are depicted in Figures 2–4. The differences in in-EI between the adolescent and college student groups are minimal, as illustrated in Figure S11–S12. For adolescents, anhedonia (D1) and breathing difficulty (A2) exhibited the highest in-EI. Heart awareness (A6) emerged as the most central symptom within in-EI for college students, while in-El for college students, while irrational fear (A7) exhibited the highest out-EI. In the elderly group, mouth dryness (A1) manifested as the symptom with the highest in-EI, while irrational fear (A7) held the highest values for both out-EI and bridge-EI symptom. Detailed comparisons of symptom centrality are provided in the Supporting Information (Figure S11–S16). Owing to the low stability of out-EI and bridge-EI rankings in the adolescent and college student groups, which did not meet the stability threshold recommended by Epskamp, Borsboom, and Fried [48], centrality rankings in these networks are not interpreted.

### 3.7. Network Comparison

The global properties of the CLPNs are shown in Table 3. The college student group exhibited the highest transitivity (0.90), followed closely by the adolescent group (0.84), whereas the elderly group demonstrated lower transitivity (0.71). This indicates that the symptom networks for the college students and adolescents were more clustered compared to the elderly group. Regarding the APL, both adolescent (1.37) and college student (1.34) networks had shorter APLs, denoting more efficient symptom transmission, in contrast to the elderly group (1.65). All three groups had a small-world index greater than 1, signifying small-world properties in each CLPN, with the index being notably higher for older adults (1.94) than for adolescents and college students (1.56 each).

In comparing the edges between the three networks, the Jaccard index showed 0.50 for the adolescent and college student CLPNs, 0.32 for the adolescent and elderly CLPNs, and 0.26 for the college student and elderly CLPNs. The Jaccard index between the adolescent and college student groups compared to the elderly group was below 0.5, indicating substantial differences in network structure between these groups.

## 4. Discussion

In this study, CLPN analyses were utilized to examine the longitudinal associations between depression and anxiety symptoms across three distinct age cohorts of Chinese individuals: adolescents, college students, and older adults. The analyses revealed variances in the network structures (CLPNs) across these age groups, with particular emphasis on the role of irrational fear and somatic anxiety as pivotal in the comorbidity of depression and anxiety.

In this study, both college students and adolescents demonstrated much higher levels of depression and anxiety compared to older adults. This aligns with earlier findings that reported lower prevalence of mood disorders in older adults [54]. The rates of depression and anxiety among adolescents and older adults were consistent with previous studies, whereas college students exhibited higher rates [55–58]. This might be linked to the more stringent pandemic-related measures commonly implemented at Chinese universities, combined with the increased exposure to pandemic-related information via the Internet, potentially heightening depression and anxiety among college students [59].

Regarding the overall characteristics of the CLPNs, it was observed that the elderly group exhibited more pronounced small-world network properties compared to the adolescent and college student groups. Small-world networks are typically defined by their scale-free structures, characterized by a limited number of nodes with extensive connections, leading to a network where central nodes are highly interconnected, whereas peripheral nodes have fewer connections [60, 61]. The implication of this structure is that central symptoms, such as irrational fear, can have a disproportionate influence on the network's overall functionality. This pattern was evident in the elderly group's CLPN (Figure 1), where fluctuations in central symptoms could significantly affect the group's overall emotional well-being.

The college student cohort demonstrated the highest transitivity and the shortest APL among the groups, with the adolescent cohort showing similar tendencies. These findings indicate a more interconnected relationship between depression and anxiety symptoms within these younger groups. Network diagrams revealed a homogeneous distribution of symptoms connections, encompassing all symptoms. This pattern suggests a “tangled” interrelationship between depression and anxiety symptoms in young populations, where no singular core symptom prevails, but rather each symptom can potentially propagate through the network, influencing others. This observation aligns with recent cross-lagged network analysis of depression and anxiety in adolescents, which also highlighted the close interconnection between these symptoms [33].

From a symptomatic perspective, the role of irrational fear differed across the networks of adolescents, college students, and older adults. Specifically, within the adolescent network, irrational fear was predominantly predicted by feelings of worthlessness, acting as the initial point in the network's strongest bridge connection. For college students, irrational fear was identified as the starting point for the most robust network connections. Among older adults, irrational fear concluded all three of the strongest edges, including the paramount bridge edge. Concerning symptom centrality, irrational fear exhibited the highest out-EI in both college student and older adult network.

Irrational fear, defined as “I felt scared without any good reason,” epitomizes a pervasive state of fearfulness [40]. Research indicates that depression associates with an elevated fear of evaluation [62, 63], which often stems from diminished self-esteem [64, 65]. Adolescents, in particular, are thought to be more sensitive to external perceptions of their self-image compared to other age groups [66]. This heightened self-awareness may link closely to their increased experiences of fear [67, 68]. In the networks of both college students and older adults, irrational fear frequently predicted somatic anxiety responses, such as dry mouth, breathing difficulties, and accelerated heart rate, underscoring the connection between fear and physical anxiety symptoms [69, 70]. During the COVID-19 pandemic, older adults reported pronounced death anxiety, characterized by an intense and unsettling fear of death [71]. Furthermore, existential worries, including fear of death and a sense of meaninglessness, are posited to significantly contribute to depressive states [72].

In addition, anhedonia, breathing difficulty, heart awareness, and mouth dryness were identified as the highest in-EI points in the CLPNs of adolescent, college student, and older, respectively, suggesting their susceptibility to other symptoms. Notably, these symptoms are predominantly physical manifestations of anxiety. According to the tripartite model of depression, negative affect is a common factor in both depression and anxiety, while physiological hyperarousal is typically associated with anxiety [15]. However, some studies suggest that physiological hyperexcitability is a shared feature in both depression and anxiety [20, 73], a perspective supported by the current study's findings. Clinically, this relationship implies that somatic symptoms' presence warrants simultaneous consideration and screening for potential underlying emotional factors.

Concerning network edges, among adolescents, the most prominent edges within the CLPN were from meaninglessness to anhedonia in depression, and the most significant bridge edges were from worthlessness in depression to irrational fear in anxiety, and from meaninglessness to panic. These edges stemmed primarily from depressive symptoms, suggesting that in adolescent comorbidity, depressive symptoms may predict the emergence of anxiety symptoms (Figure 1a). This aligns with some previous research indicating that depression symptoms often precede anxiety symptoms [74, 75], although other studies have reported anxiety preceding depression in adolescents, with variations possibly attributable to the specific types of anxiety disorders [76, 77]. In the college student population, the strongest edge shifted from irrational fear (anxiety) to heart awareness (anxiety), while the most notable bridge transitioned from difficulty initiating actions (depression) to fear of embarrassment (anxiety). Network diagrams for this group did not show a clear sequence between depression and anxiety symptoms (Figure 1b). For older adults, the strongest edges consistently originated from anxiety symptoms (irrational fear, Figure 1c), suggesting a developmental pattern where the predictable symptoms transition from depressive to anxiety-related as age increases. This is consistent with findings that certain anxieties, like fear of falling, become more prevalent in later life and that anxiety disorders can precede depression in older age [78–80]. Although previous findings on the bidirectional association of depression and anxiety have yielded mixed results, systematic cross-age group comparisons remain scarce [81–83]. Thus, the developmental patterns suggested in this study are preliminary and necessitate further empirical validation.

During the COVID-19 outbreak, several studies explored the association between fear of the virus, anxiety, and depression [84–86]. The findings of these studies, particularly the review by Çıkrıkçı, Çıkrıkçı, and Griffiths [85], align with our results, indicating that fear of COVID-19 could influence depression via anxiety. Therefore, the prominence of irrational fear identified in the present study might be linked to the pervasive fearfulness induced by the pandemic during our data collection period.

This study is a pioneering effort in longitudinally exploring the relationship between depression and anxiety symptoms while also conducting cross-age comparisons using a large sample spanning three distinct age groups. The findings can be summarized in three key points. First, network structures differed across age groups, with adolescents and college students showing a closely intertwined relationship between depression and anxiety symptoms, whereas older adults exhibited a stronger small-world structure dominated by irrational fear. As age increased, anxiety symptoms appeared more predictive. Second, irrational fear emerged as a central symptom across all age groups, particularly during pandemic conditions. Third, the study underscored that somatic anxiety symptoms often correlated with other psychological symptoms.

At the theoretical level, our study offers an exploratory look into the development of depression and anxiety comorbidity patterns across age groups, drawing from network theory. Based on this perspective, we propose that as individuals age, their capacity for emotional regulation tends to increase, causing depression and anxiety symptoms to evolve from a tightly connected cluster into relatively independent symptom networks. In younger populations, a change in one symptom may signal an amplification or reduction of the overall emotional response. However, in older adults, only a few core symptoms are likely to influence others, confining fluctuations to a narrower range. This hypothesis is consistent with prior research suggesting that older individuals exhibit greater selective control over their emotions and experience fewer mood fluctuations [87–89]. Nevertheless, this remains a preliminary hypothesis, and extensive empirical research will be necessary to further substantiate it. Additionally, our findings highlight the important role that somatic symptoms play in both anxiety and depression, reinforcing the view that these symptoms are strongly related to both [20, 73].

In terms of clinical practice, these results underscore the need for age-specific intervention strategies. For adolescents, interventions targeting depressed mood and cognitive symptoms, such as cognitive behavioral therapy, may be especially beneficial [90, 91]. For college students and older adults, strategies that address heightened emotional responses, such as worry and fear, including mindfulness meditation [92, 93], might prove more helpful. Furthermore, the study suggests that somatic symptoms, which frequently appear as manifestations of psychological distress, should not be overlooked. Clinicians are encouraged to assess for underlying emotional issues when patients present with physical symptoms like dry mouth or breathing difficulties, as these may signal deeper emotional concerns, particularly in older adults.

However, the study is not without limitations. The age cohorts were not uniformly sampled in terms of location and timing, which might have affected the network characteristics due to variable pandemic impacts. Future studies could further explore the comorbidity network in different age groups without pandemic background. Also, the 6-month time interval and the use of only two time points limited the depth of our longitudinal analysis. Future research could extend this by incorporating microdynamic analyses, increasing the study interval, and adding more data collection waves. Despite our use of the maximum expectation approach to missing values, attrition bias may still result from more severe baseline depression and anxiety among dropped college students and older age among quit adolescents.

Another limitation is the lack of exploration of comorbidity networks in adults in the 30–50 age group. Given that this age group has to cope with multiple stressors such as work and parenting, further research on their depression–anxiety comorbidity networks is warranted. Finally, in terms of specific outcomes, this study did not perform rigorous statistical comparisons across networks to quantify differences. The relatively low stabilities of CLPN out-EI and bridge-EI in adolescents and college students necessitate further validation across groups to enrich the understanding of symptom centrality.

## Figures and Tables

**Table 1:** Reliability for each administration of the scale (Cronbach's alpha).

Group	Depression		Anxiety	
	T1	T2	T1	T2
Adolescent	0.84	0.83	0.87	0.85
College student	0.90	0.92	0.89	0.91
Elderly	0.75	0.77	0.74	0.64

**Table 2:** Descriptive statistics and demographic data.

	Time	Adolescent		College student		Elderly	
		*M* (SD)	Prevalence	*M* (SD)	Prevalence	*M* (SD)	Prevalence
Depression	T1	5.57 (6.72)	21.60%	9.72 (7.86)	51.80%	2.92 (4.14)	9.10%
	T2	7.38 (7.45)	30.10%	9.79 (7.23)	55.60%	2.04 (3.31)	5.20%

Anxiety	T1	7.25 (7.27)	39.70%	10.31 (7.62)	61.00%	5.65 (5.09)	24.60%
	T2	9.28 (7.75)	53.40%	10.01 (7.16)	61.90%	4.41 (3.94)	20.30%

Age	T1	15.98 (0.97)		19.94 (1.33)		85.19 (8.75)	
	T2	16.07 (1.16)		20.33 (1.10)		85.00 (7.98)	

Gender	T1	45.8% male		58.9% male		46.5% male	
	T2	45.1% male		57.1% male		46.4% male	

**Table 3:** Global characteristics of CLPNs.

Group	Transitivity	APL	Small-world index
Adolescent	0.84 (0.58)	1.37 (1.48)	1.56
College student	0.90 (0.62)	1.34 (1.45)	1.56
Elderly	0.71 (0.38)	1.65 (1.73)	1.94

*Note:* Represented in parentheses are the results computed for comparable random networks (with the same number of points and edges). A network is considered to have small-world feature if its APL is closer to random network and its transitivity is much higher.

Abbreviations: APL, average path length; CLPNs, cross-lagged panel networks.

## Data Availability

The data that support the findings of this study are openly available in the data of DASS at three age stages at https://data.mendeley.com/datasets/c2mxwrgc4d/3, reference number DOI: 10.17632/c2mxwrgc4d.3.

## Supporting Information

Supporting Information Supporting information includes Table S1 and Figures S1–S16 of the nonprimary results of this study. Specific image content is cited in the Results section.
