# Supplementary material for: Longitudinal Associations Between Anxiety and Depressive Symptoms in Adolescence, Early Adulthood, and Old Age: Cross-Lagged Panel Network Analyses
Source: Depress Anxiety. 2024 Nov 29;2024:6205475. doi: 10.1155/da/6205475 (PMC11919059; doi:10.1155/da/6205475)
Supplement: Supporting Information — includes Table S1 and Figures S1–S16 of the nonprimary results of this study. Specific image content is cited in the Results section. [file 6205475.f1.docx]

**Table S1.** Baseline difference tests for stayed and dropped out subjects

|  | Subject category | N | M | SD | *t* | *p* |
| --- | --- | --- | --- | --- | --- | --- |
| Adolescent | | | | | | |
| depression | Stayed | 1154 | 2.74 | 3.28 | -1.54 | 0.12 |
|  | dropped out | 104 | 3.27 | 4.14 |  |  |
| anxiety | Stayed | 1154 | 3.61 | 3.56 | -0.42 | 0.67 |
|  | dropped out | 104 | 3.77 | 4.35 |  |  |
| age | Stayed | 1154 | 15.95 | 0.97 | -4.55 | >0.001 |
|  | dropped out | 104 | 16.39 | 0.92 |  |  |
| College student | | | | | | |
| depression | Stayed | 907 | 3.65 | 3.41 | -4.35 | >0.001 |
|  | dropped out | 212 | 4.80 | 3.63 |  |  |
| anxiety | Stayed | 907 | 4.98 | 3.77 | -3.15 | 0.00 |
|  | dropped out | 212 | 5.90 | 3.89 |  |  |
| age | Stayed | 907 | 19.95 | 1.36 | 0.80 | 0.42 |
|  | dropped out | 212 | 19.87 | 1.19 |  |  |
| Elderly | | | | | | |
| depression | Stayed | 460 | 1.51 | 2.12 | 1.21 | 0.23 |
|  | dropped out | 88 | 1.21 | 1.77 |  |  |
| anxiety | Stayed | 460 | 2.83 | 2.56 | 0.21 | 0.83 |
|  | dropped out | 88 | 2.77 | 2.47 |  |  |
| age | Stayed | 460 | 84.97 | 8.00 | -1.32 | 0.19 |
|  | dropped out | 88 | 86.32 | 11.86 |  |  |

**Figure S1.** Bootstrapped 95% confidence intervals around each edge weight for T1 → T2 adolescent network.


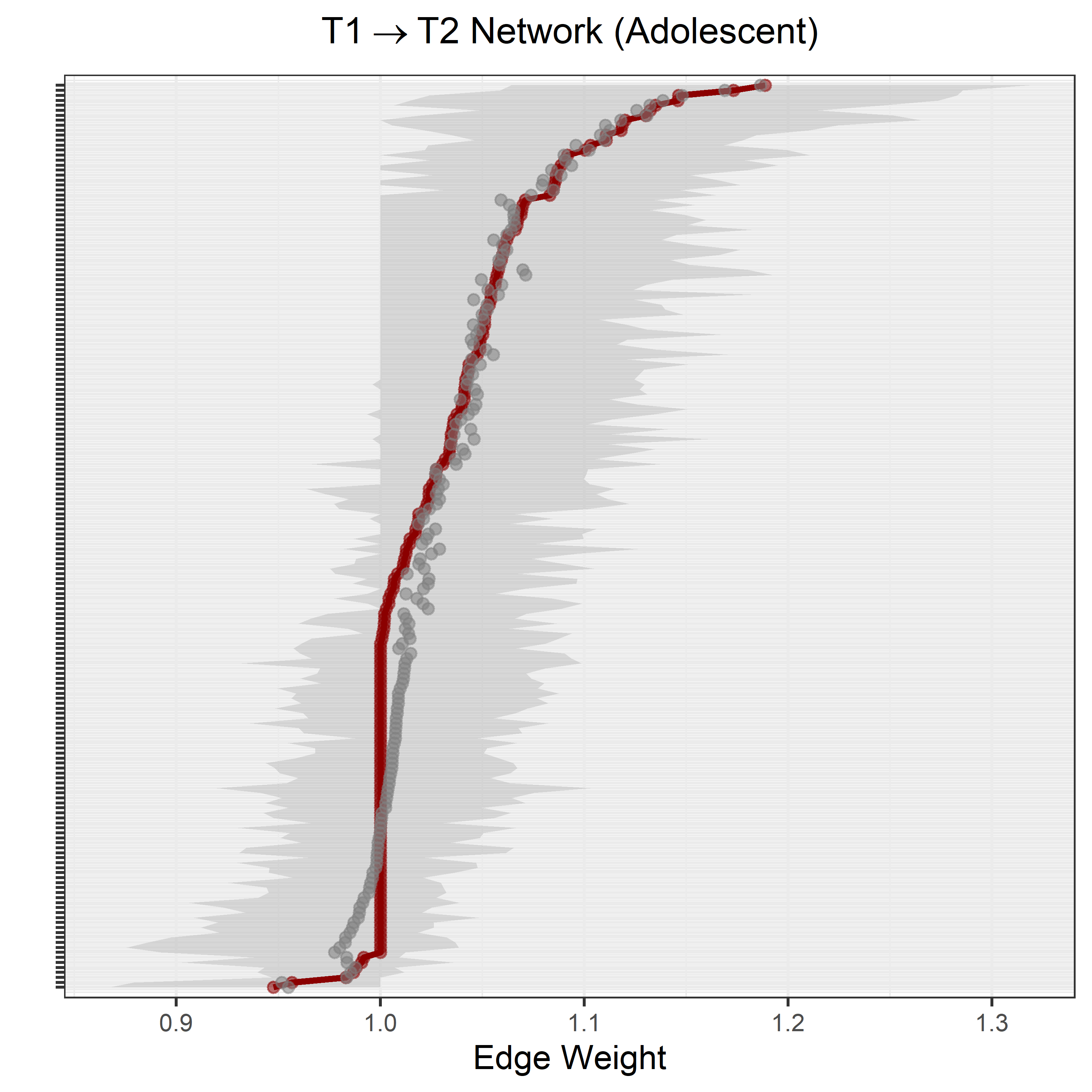


*Note.* Red lines indicate the edge weight in the estimated sample network.

**Figure S2** Bootstrapped 95% confidence intervals around each edge weight for T1 → T2 college student network.


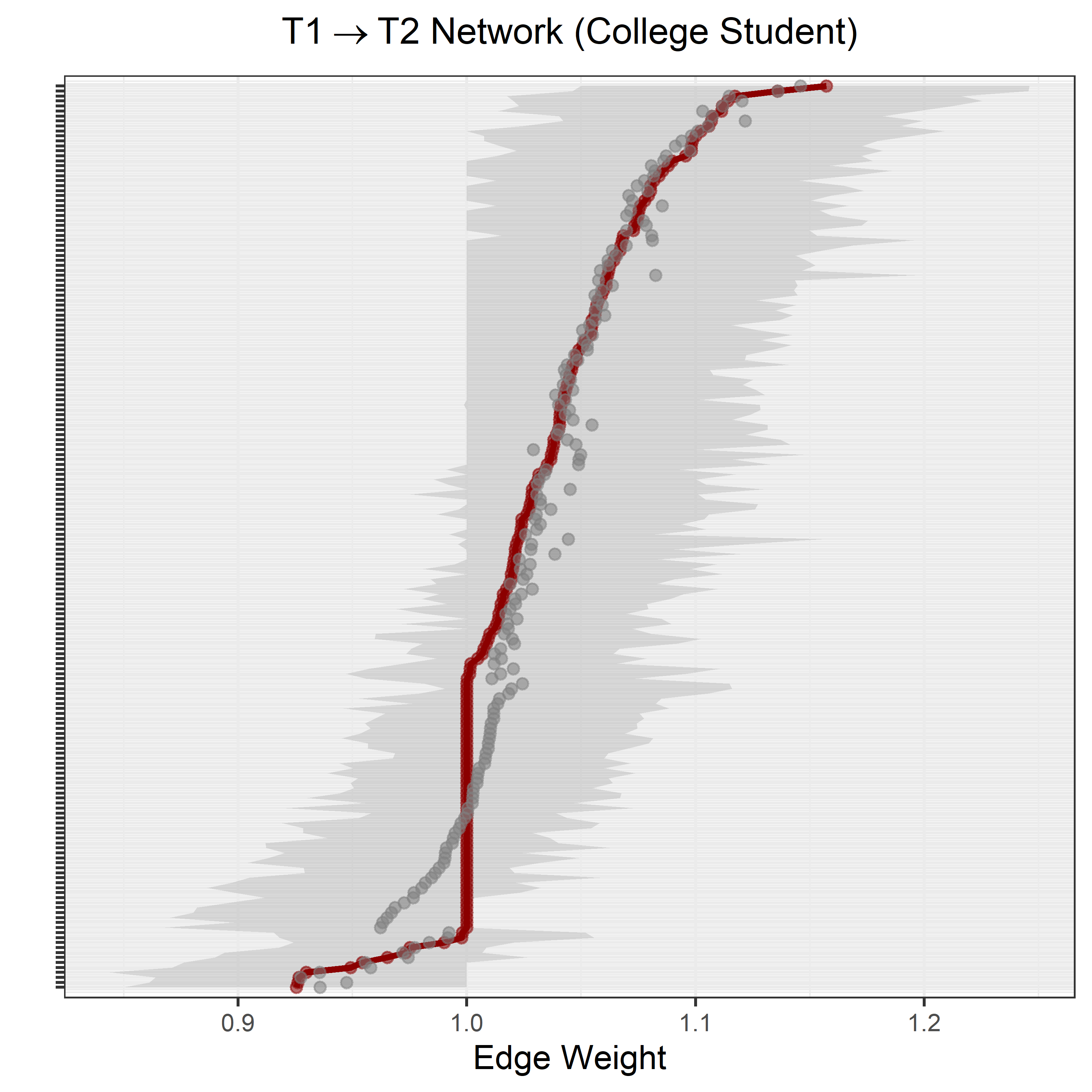


*Note.* Red lines indicate the edge weight in the estimated sample network.

**Figure S3** Bootstrapped 95% confidence intervals around each edge weight for T1 → T2 elderly network.


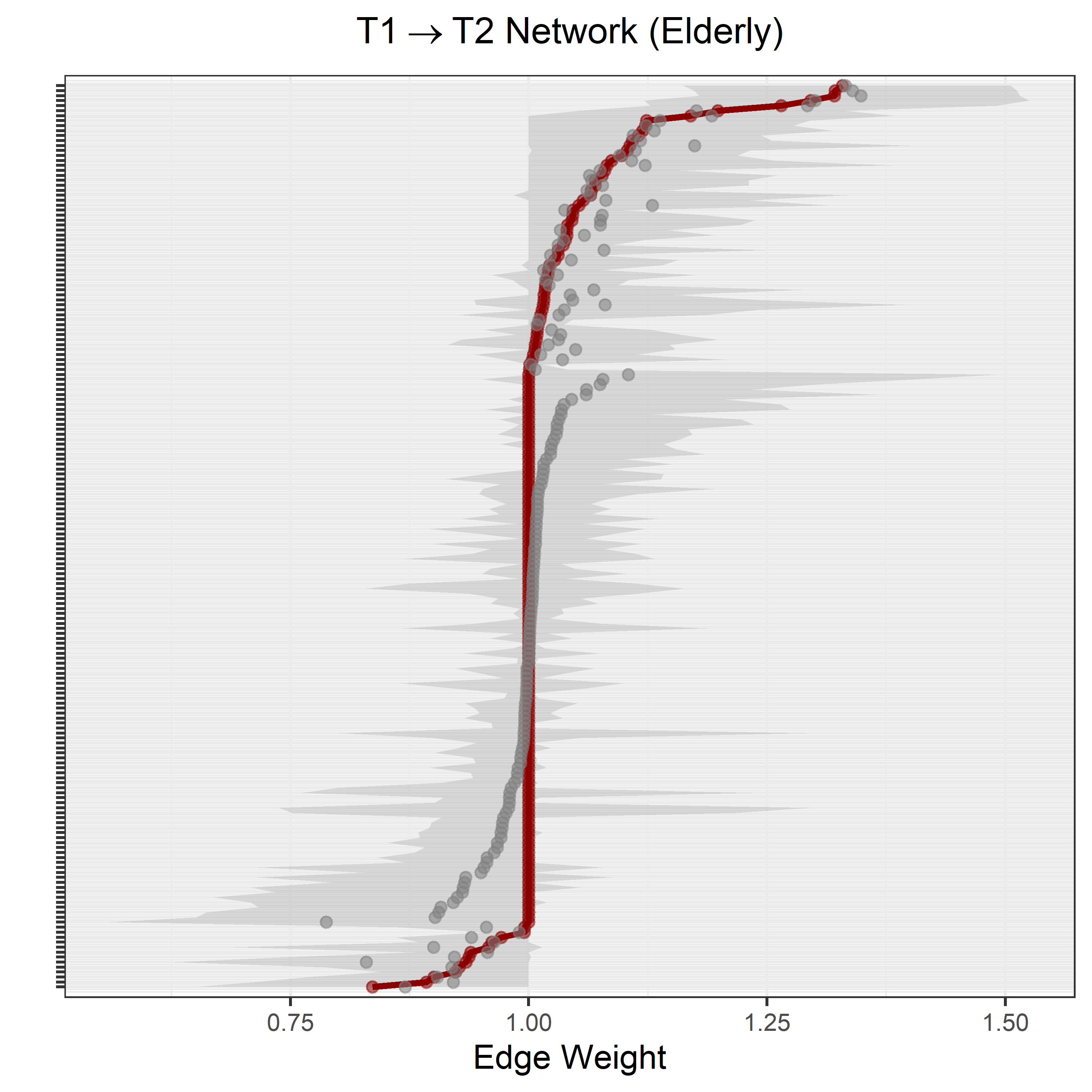


*Note.* Red lines indicate the edge weight in the estimated sample network.

**Figure S4.** Stability of centrality measures for T1 → T2 adolescent networks.


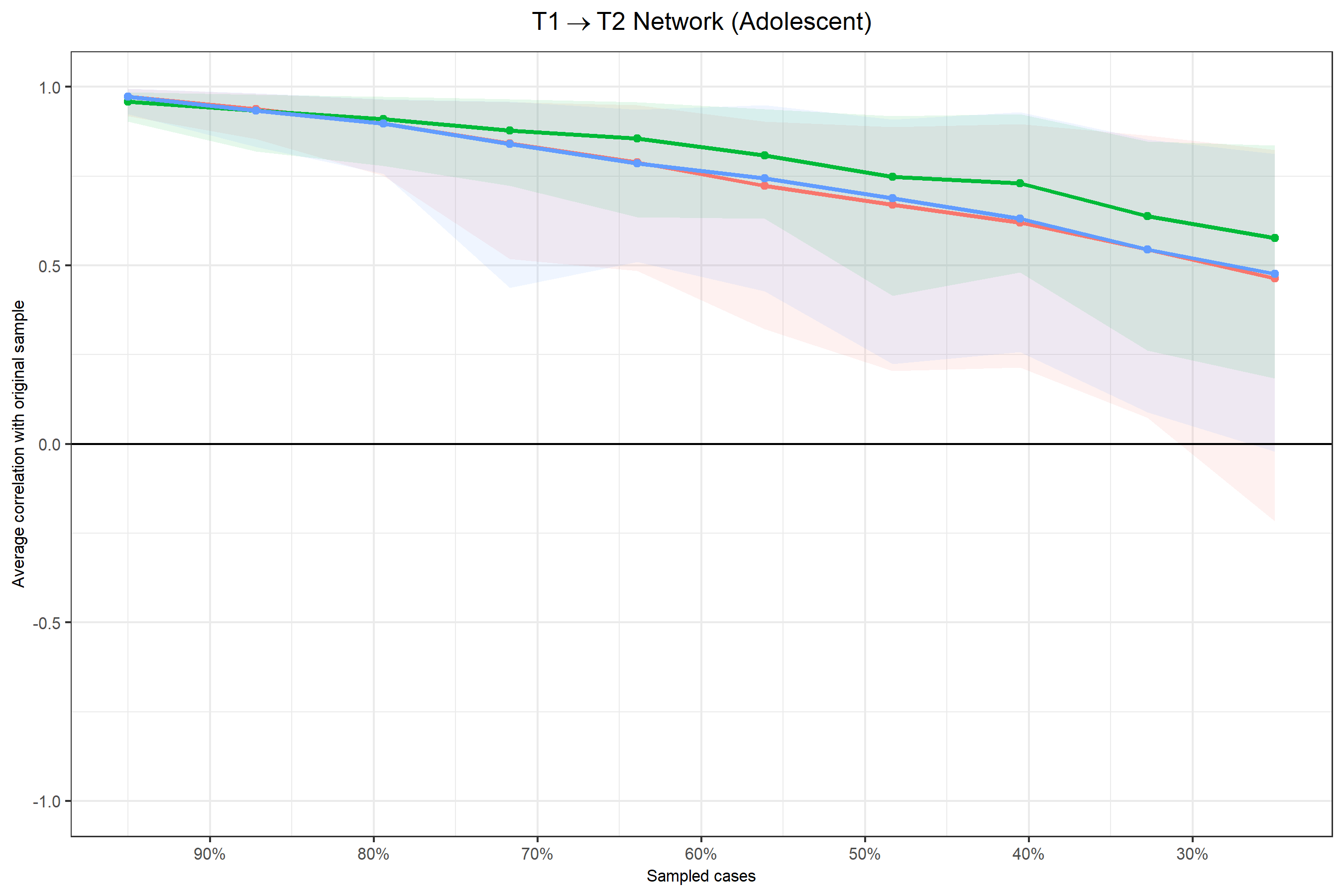


**Figure S5.** Stability of centrality measures for T1 → T2 college student networks.


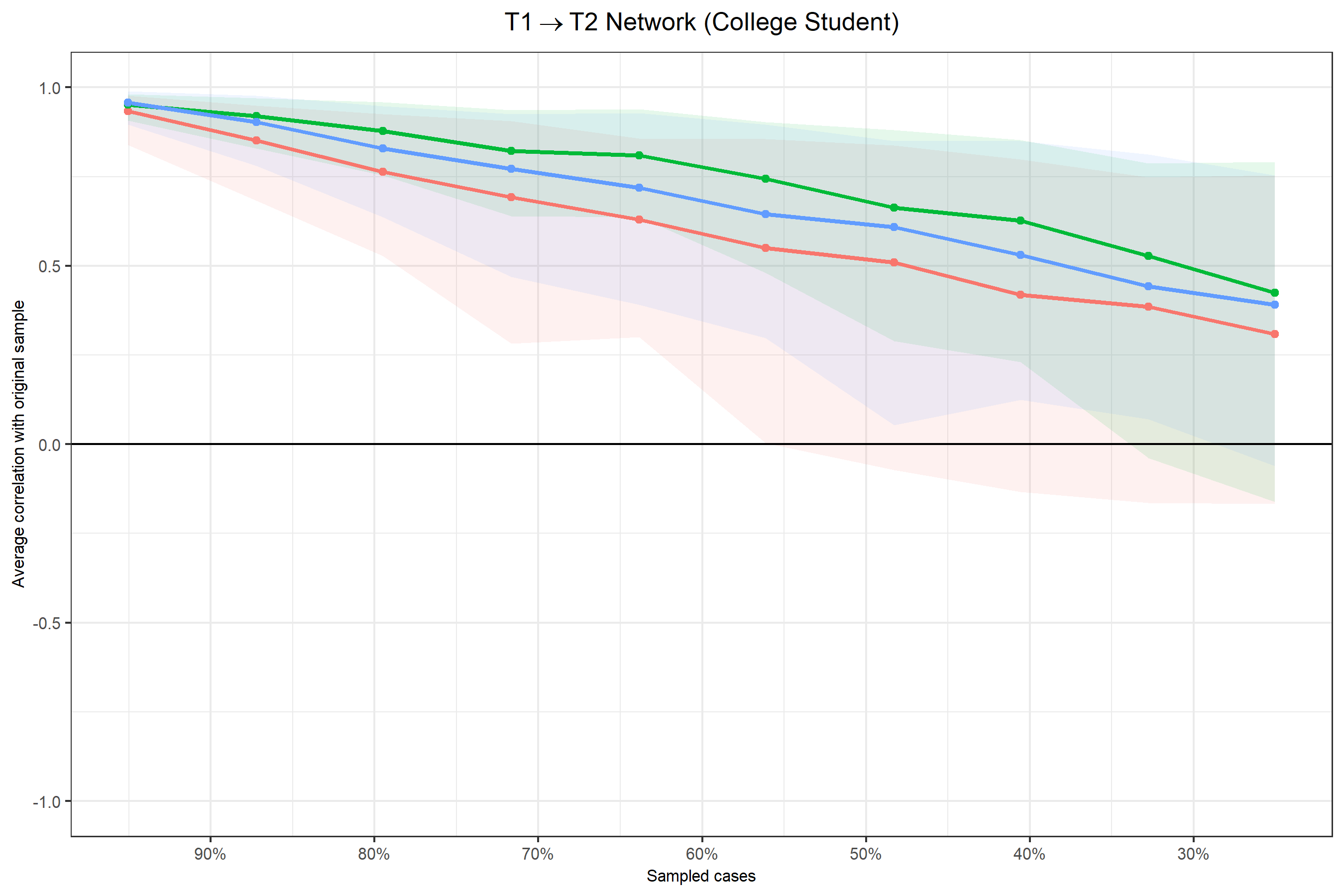


**Figure S6.** Stability of centrality measures for T1 → T2 college student networks.


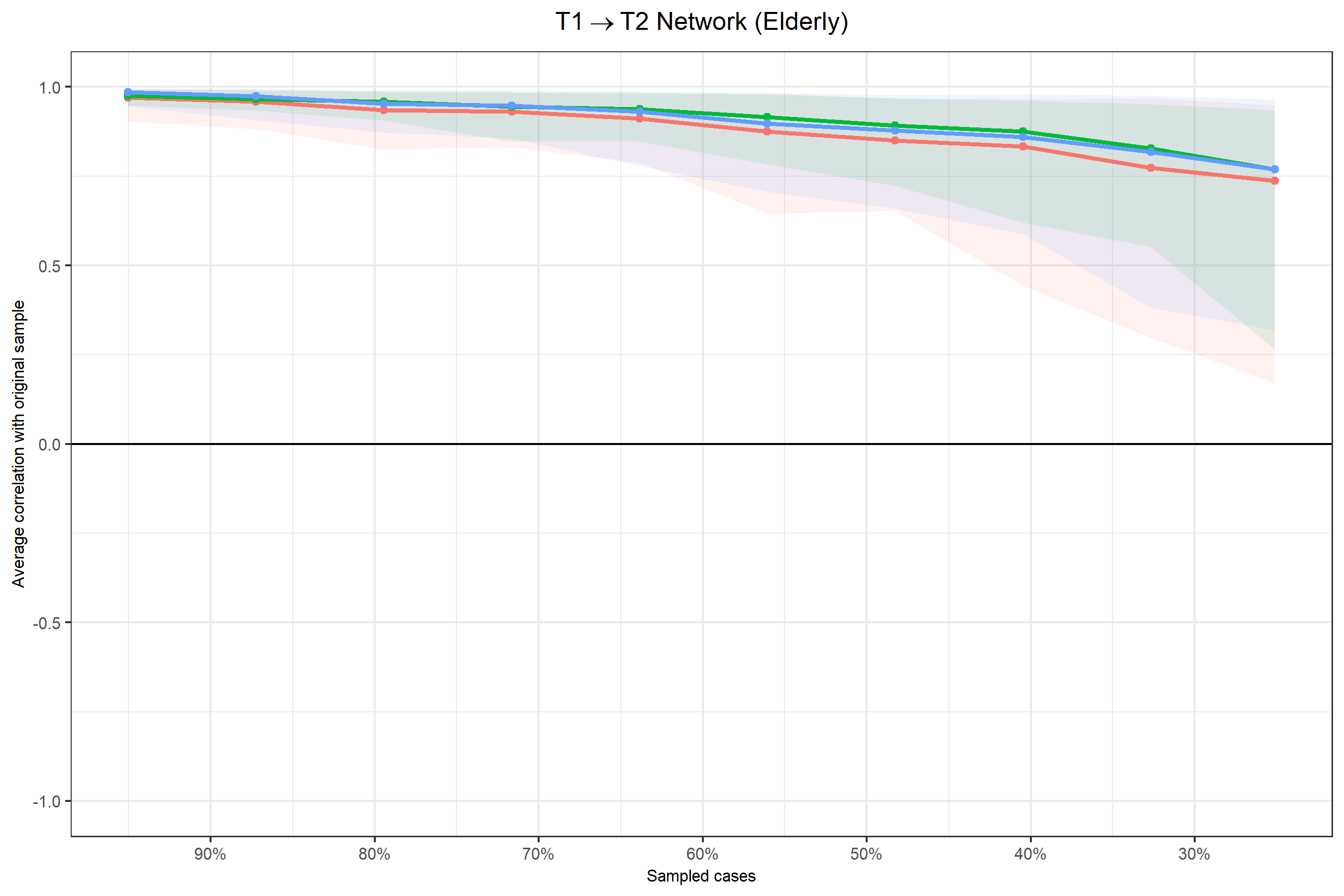


**Figure S7.** Odds ratio of autoregressive edges for three networks.


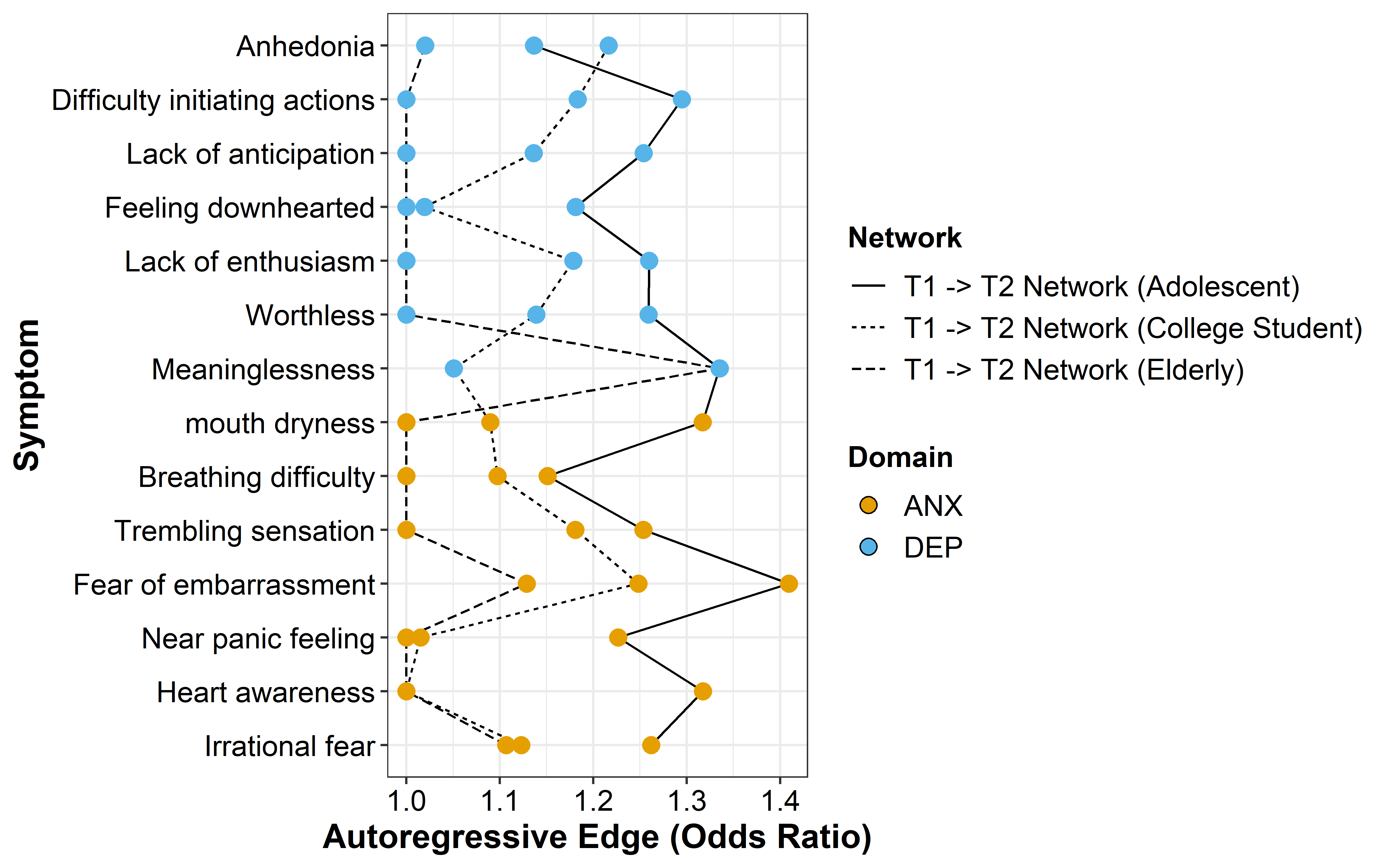


*Note.* Mean value of the odds ratio at autoregressive edges: *M* _adolescent_= 1.12; *M* _college student_= 1.26;

*M* _elderly_= 1.05

**Figure S8.** Edge weight difference tests for the T1 → T2 adolescent network.





*Note.* Black boxes indicate edges that significantly differ (p < .05), and gray boxes indicate edges that not significantly differ.

**Figure S9.** Edge weight difference tests for the T1 → T2 college student network.





*Note.* Black boxes indicate edges that significantly differ (p < .05), and gray boxes indicate edges that not significantly differ.

**Figure S10.**  Edge weight difference tests for the T1 → T2 college student network.





*Note.* Black boxes indicate edges that significantly differ (p < .05), and gray boxes indicate edges that not significantly differ.

**Figure S11.** In-expected influence difference tests for the T1 → T2 adolescent network.


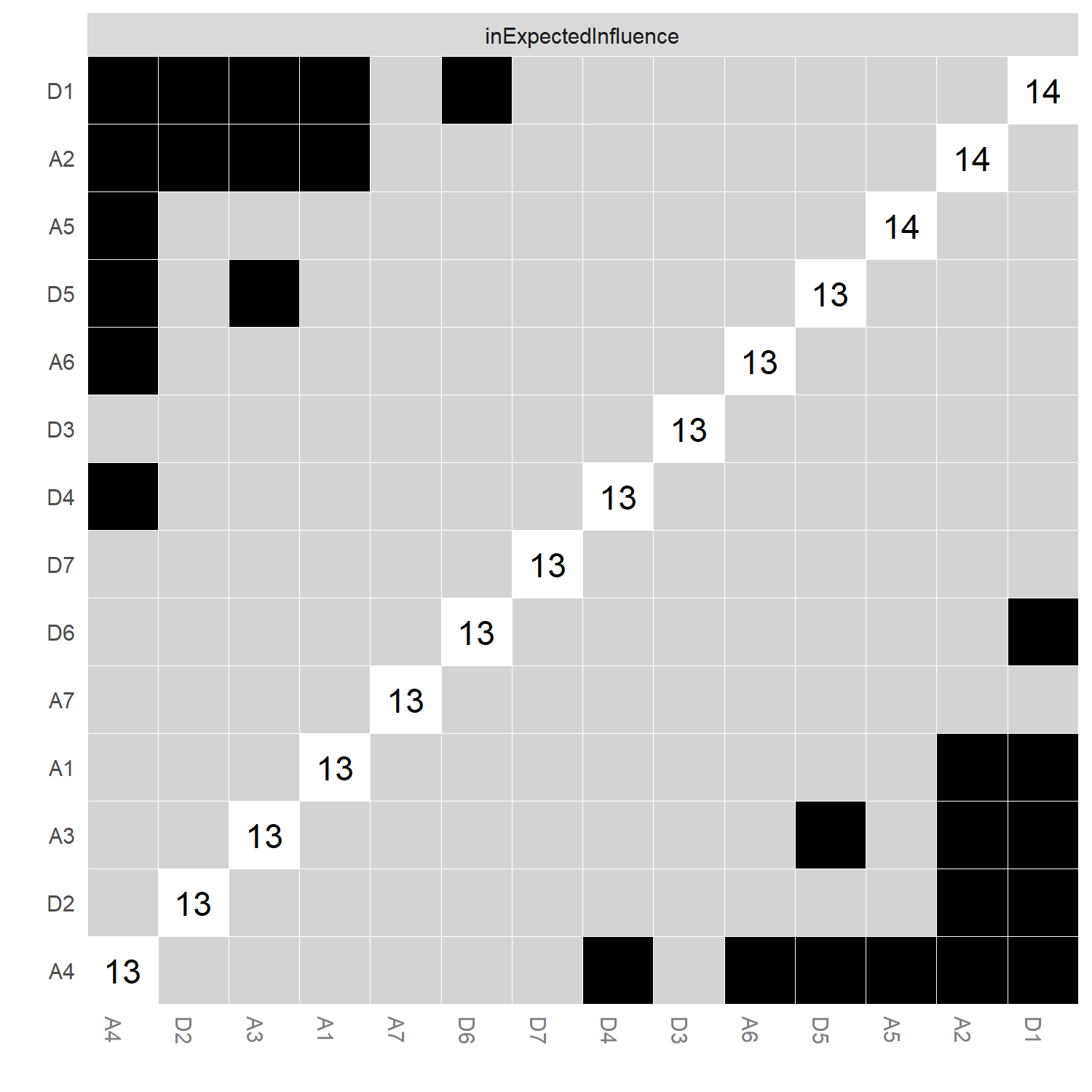


*Note.* Black boxes indicate symptoms that significantly differ in centrality (p < .05), and gray boxes indicate symptoms whose centrality does not significantly differ.

**Figure S12.** In-expected influence difference tests for the T1 → T2 college student network.


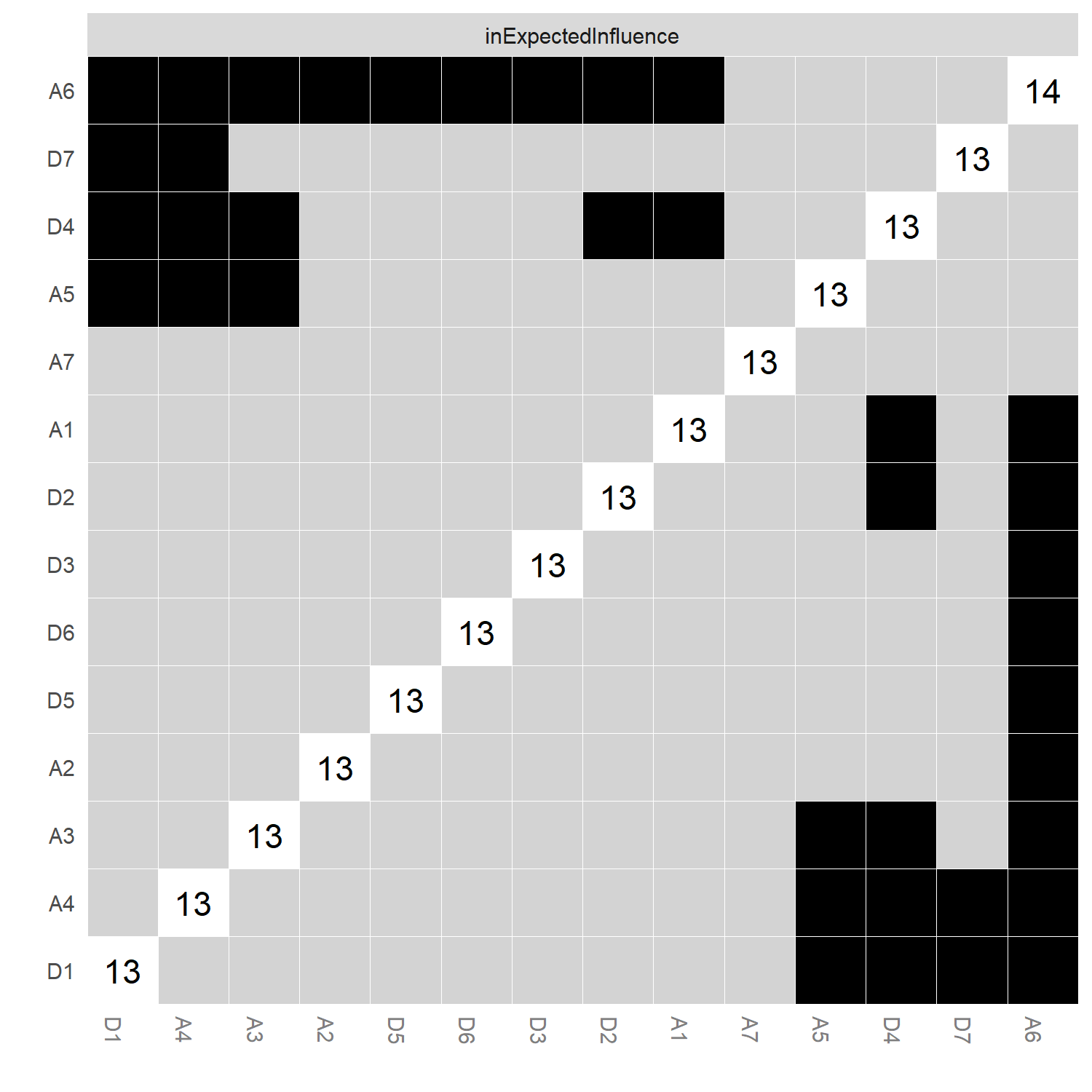


*Note.* Black boxes indicate symptoms that significantly differ in centrality (p < .05), and gray boxes indicate symptoms whose centrality does not significantly differ.

**Figure S13.** Out-expected influence difference tests for the T1 → T2 college student network.


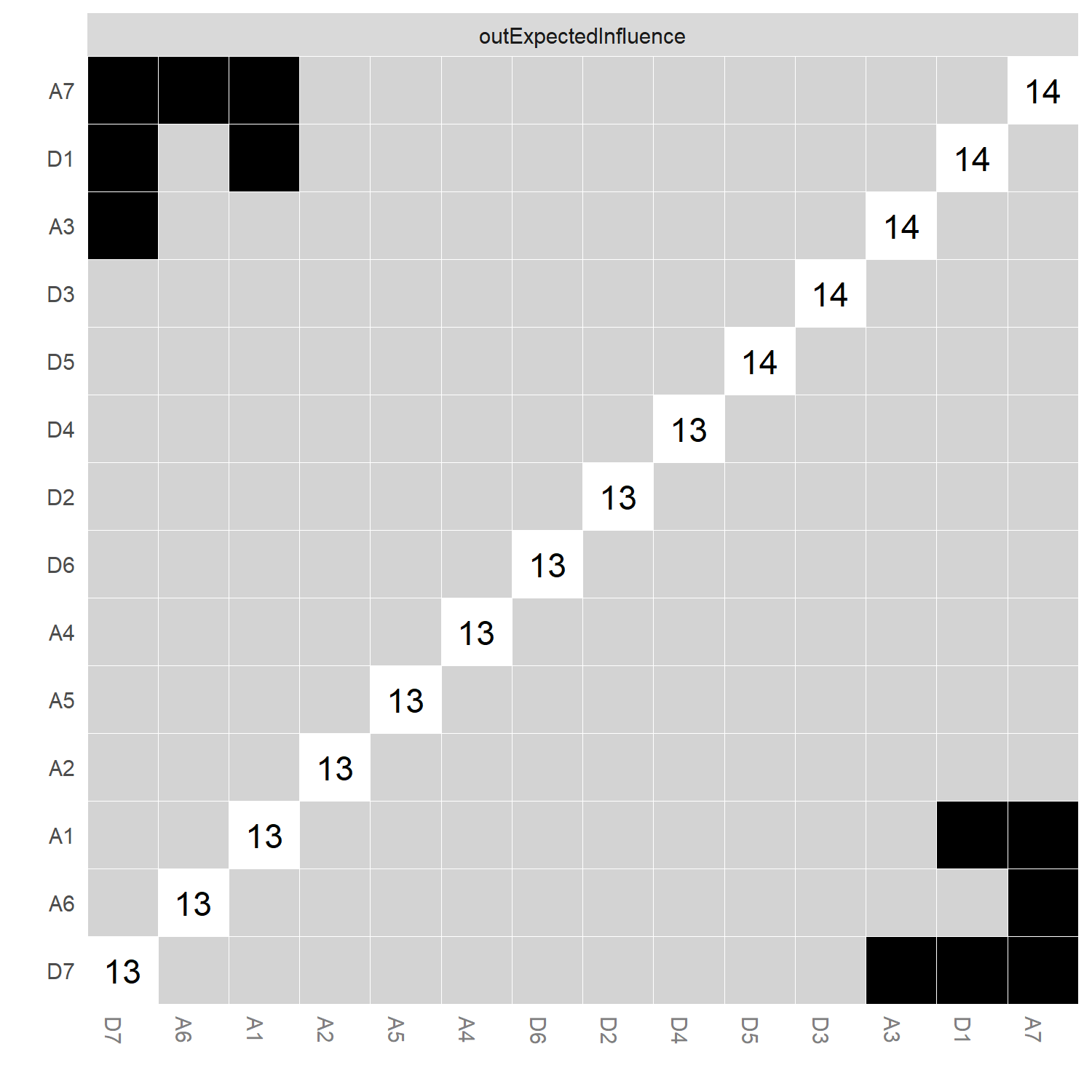


*Note.* Black boxes indicate symptoms that significantly differ in centrality (p < .05), and gray boxes indicate symptoms whose centrality does not significantly differ.

**Figure S14.** In-expected influence difference tests for the T1 → T2 elderly network.


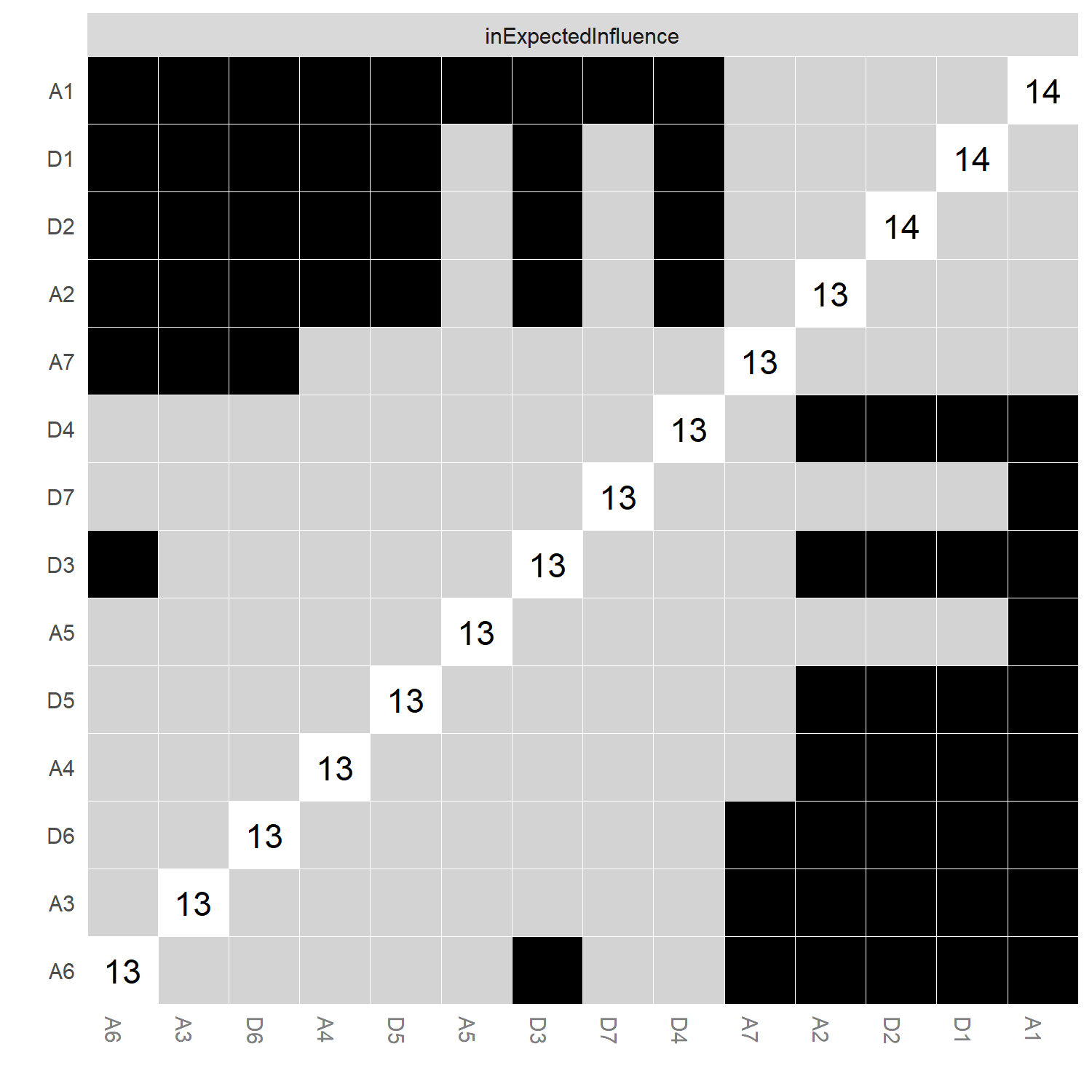


*Note.* Black boxes indicate symptoms that significantly differ in centrality (p < .05), and gray boxes indicate symptoms whose centrality does not significantly differ.

**Figure S15.** Out-expected influence difference tests for the T1 → T2 elderly network.


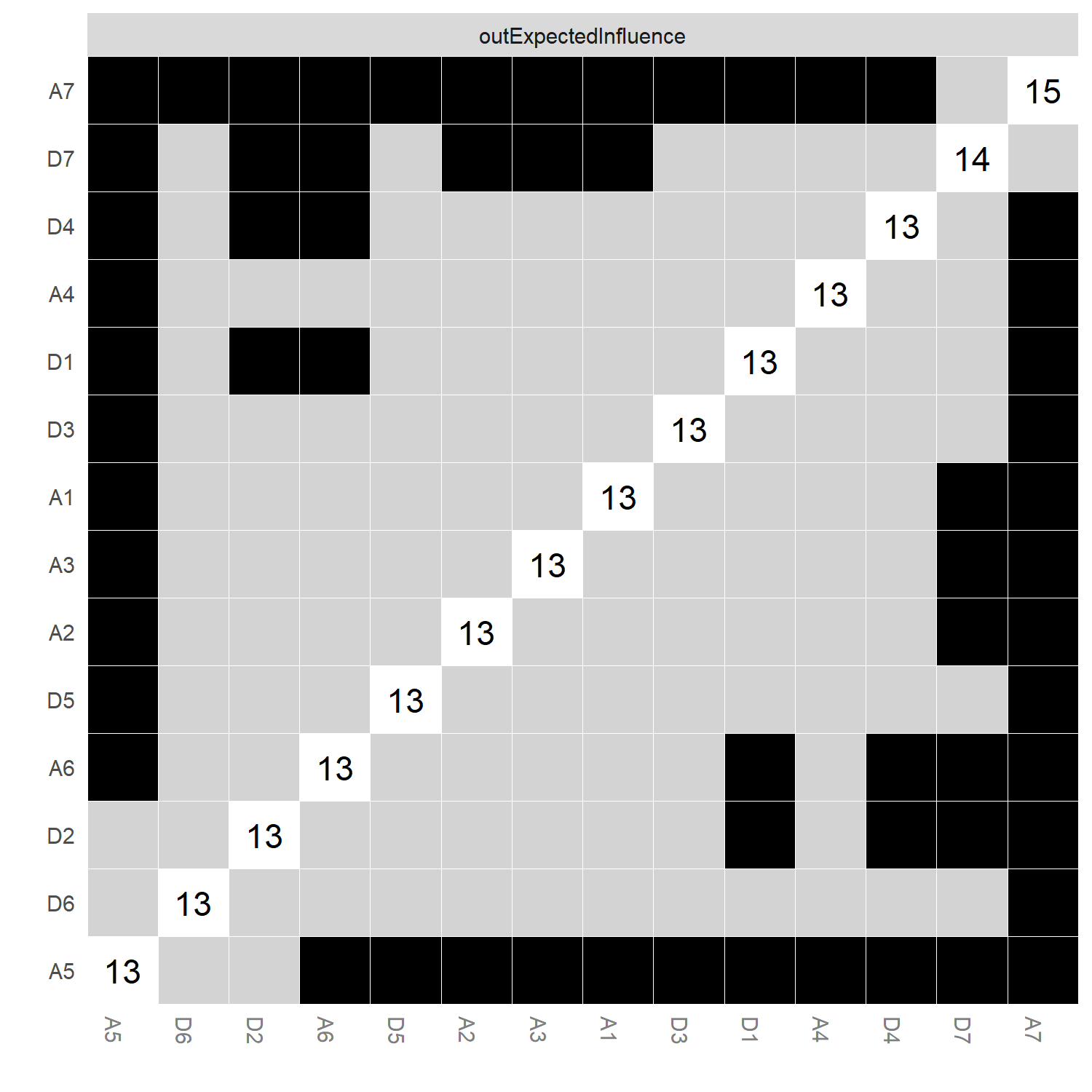


*Note.* Black boxes indicate symptoms that significantly differ in centrality (p < .05), and gray boxes indicate symptoms whose centrality does not significantly differ.

**Figure S16.** Bridge-expected influence difference tests for the T1 → T2 elderly network.


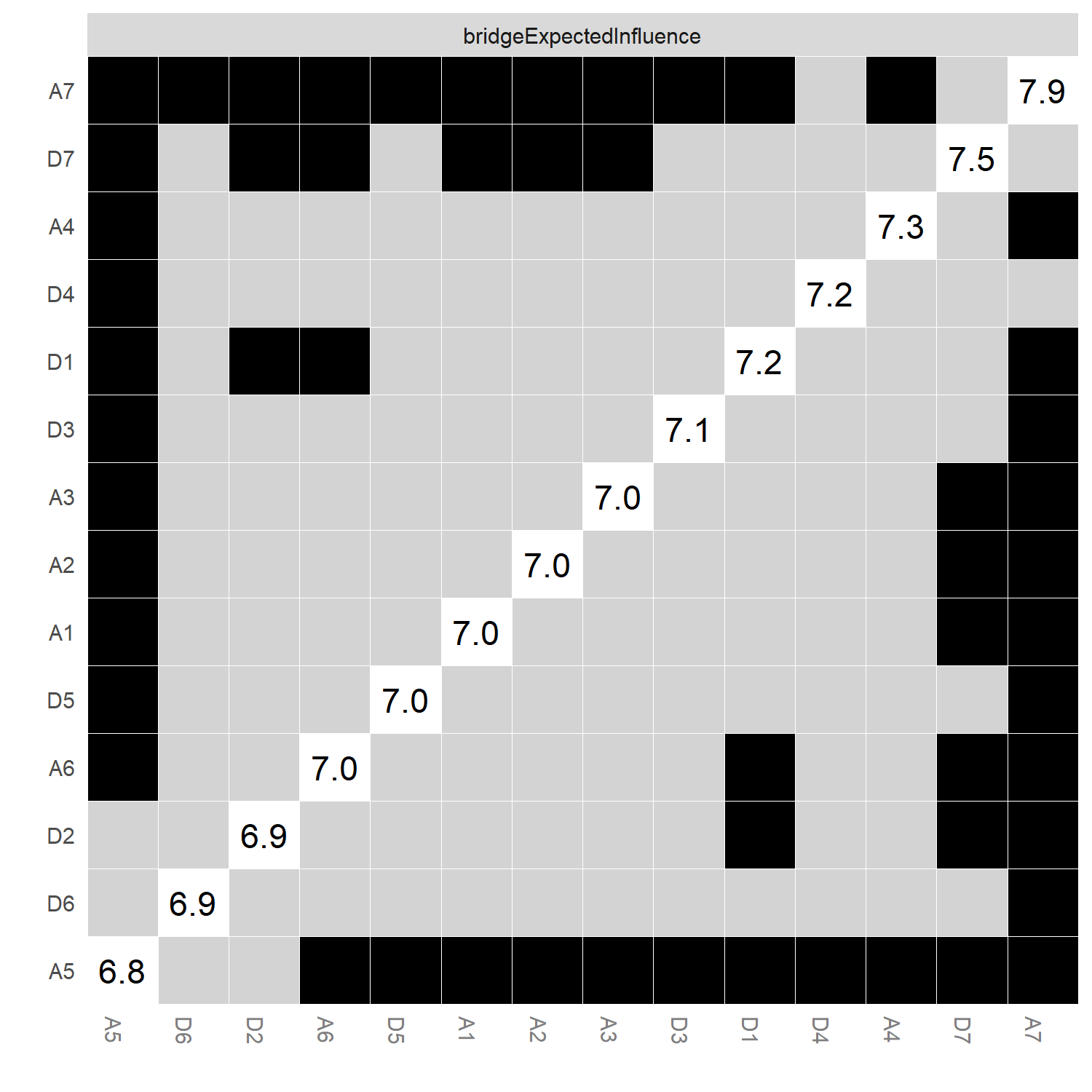


*Note.* Black boxes indicate symptoms that significantly differ in centrality (p < .05), and gray boxes indicate symptoms whose centrality does not significantly differ.
